# A third polymorph of 1,4-bis­(1*H*-benzimid­azol-2-yl)benzene

**DOI:** 10.1107/S1600536814011179

**Published:** 2014-05-24

**Authors:** Wei-Wei Fu, Yan-Fei Liang, Yang Liu, Xiao-Ming Zhu

**Affiliations:** a Key Laboratory of Functional Organometallic Materials of General Colleges and Universities in Hunan Province, Department of Chemistry and Materials Science, Hengyang Normal University, Hengyang 421008, People’s Republic of China

## Abstract

The title compound, C_20_H_14_N_4_, is a new polymorph of the previously reported structures, which were ortho­rhom­bic, space group *Pbca* [Bei *et al.* (2000). *Acta Cryst.* C**56**, 718–719] and monoclinic, space group *P*2_1_/*c* [Dudd *et al.* (2003). *Green Chem.* **5**, 187–192]. The asymmetric unit consists of two independent mol­ecules in which the dihedral angels between the central benzene ring and the outer benzimidazole ring systems are 16.81 (10) and 14.23 (10)° in one molecule and 26.09 (10) and 37.29 (10)° in the other. In the crystal, mol­ecules are linked by N—H⋯N and C—H⋯N hydrogen bonds into a tape running along the *c-*axis direction.

## Related literature   

For the synthesis of the title compound, see: Alcalde *et al.* (1992 ▶); Zhao *et al.* (2012 ▶); Zhuang *et al.* (2011 ▶). For the previously reported structures of the title compound, see: Bei *et al.* (2000 ▶); Dudd *et al.* (2003 ▶). For the structures of the title compound with solvent mol­ecules, see: Wu & Hu (2009 ▶); Su *et al.* (2011 ▶).
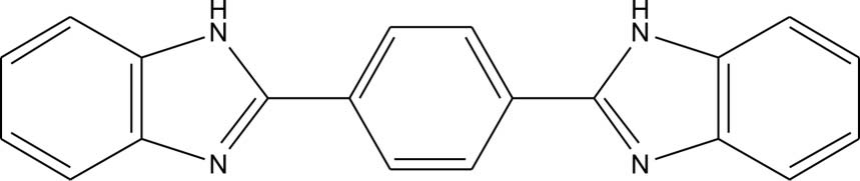


## Experimental   

### 

#### Crystal data  

C_20_H_14_N_4_
*M* *_r_* = 310.35
Monoclinic, 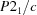

*a* = 16.196 (3) Å
*b* = 20.174 (3) Å
*c* = 9.9010 (16) Å
β = 106.733 (3)°
*V* = 3098.1 (8) Å^3^
*Z* = 8
Mo *K*α radiation
μ = 0.08 mm^−1^
*T* = 296 K
0.26 × 0.22 × 0.17 mm

#### Data collection  

Bruker APEXII CCD diffractometer
Absorption correction: multi-scan (*SADABS*; Bruker, 2001 ▶) *T*_min_ = 0.979, *T*_max_ = 0.986
15697 measured reflections
5451 independent reflections
3197 reflections with *I* > 2σ(*I*)
*R*_int_ = 0.062

#### Refinement  

*R*[*F* ^2^ > 2σ(*F* ^2^)] = 0.053
*wR*(*F* ^2^) = 0.126
*S* = 1.02
5451 reflections
450 parameters
5 restraints
H atoms treated by a mixture of independent and constrained refinement
Δρ_max_ = 0.21 e Å^−3^
Δρ_min_ = −0.21 e Å^−3^

### 

Data collection: *APEX2* (Bruker, 2007 ▶); cell refinement: *SAINT* (Bruker, 2007 ▶); data reduction: *SAINT*; program(s) used to solve structure: *SHELXS97* (Sheldrick, 2008 ▶); program(s) used to refine structure: *SHELXL97* (Sheldrick, 2008 ▶); molecular graphics: *SHELXTL* (Sheldrick, 2008 ▶) and *DIAMOND* (Brandenburg, 2008 ▶); software used to prepare material for publication: *SHELXTL* and *publCIF* (Westrip, 2010 ▶).

## Supplementary Material

Crystal structure: contains datablock(s) I, New_Global_Publ_Block. DOI: 10.1107/S1600536814011179/is5361sup1.cif

Structure factors: contains datablock(s) I. DOI: 10.1107/S1600536814011179/is5361Isup2.hkl

Supporting information file. DOI: 10.1107/S1600536814011179/is5361Isup3.cml

Additional supporting information:  crystallographic information; 3D view; checkCIF report

## Figures and Tables

**Table 1:** Hydrogen-bond geometry (Å, °)

*D*—H⋯*A*	*D*—H	H⋯*A*	*D*⋯*A*	*D*—H⋯*A*
N2—H2⋯N5	0.91 (2)	2.06 (2)	2.947 (3)	164 (2)
N4—H4⋯N3^i^	0.93 (2)	1.91 (2)	2.837 (3)	172 (2)
N6—H6⋯N1^ii^	0.92 (2)	2.00 (2)	2.910 (3)	171 (2)
N8—H8⋯N7^i^	0.90 (2)	2.15 (2)	3.041 (3)	174 (2)
C12—H12⋯N3^i^	0.93	2.57	3.396 (3)	148

Symmetry codes: (i) 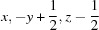; (ii) 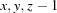.

**Fig. 1.**
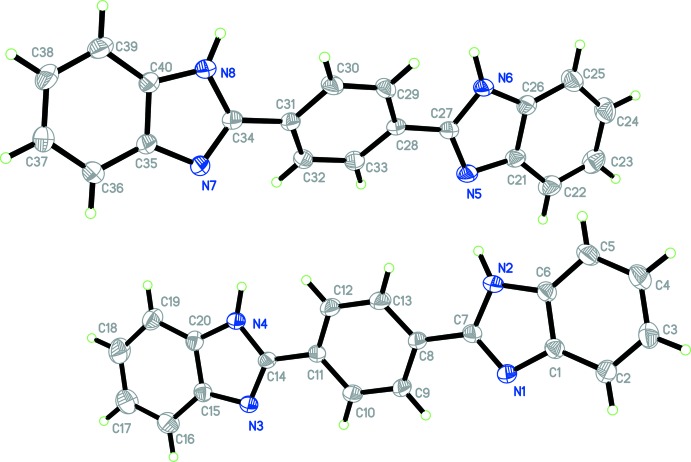
An ORTEP drawing for the asymmetric unit of the title compound with displacement ellipsoids drawn at the 30% probability level.

**Fig. 2.**
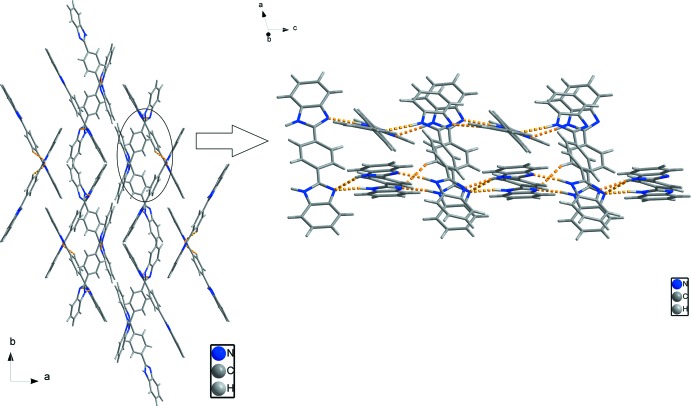
A packing diagram of the title compound viewed along c direction. The hydrogen bonds are highlighted by dashed lines.

## supplementary crystallographic information

### 1. Comment

Benzimidazole and their derivatives have been widely researched for their potential applications in medicinal chemistry, biochemistry and material chemistry. 1,4-bis(benzimidazol-2-yl)benzene has been synthesized with many methods in different groups. Zhuang have synthesized 1,4-bis(benzimidazol-2-yl)benzene with microwave method (Zhuang *et al.*, 2011). Zhao have synthesized it using phosphoric acid as a catalyst (Zhao *et al.*, 2012) instead of polyphosphoric acid which are commonly used in synthesis of benzimidazole (Alcalde *et al.*, 1992). Its crystal structure has been determined by Bei *et al.* (2000) and Dudd *et al.* (2003). Recently, its crystal structures with solvent molecules DMF or methanol have also been reported (Wu & Hu, 2009; Su *et al.*, 2011). Here, we report a new crystal structure of 1,4-bis(benzimidazol-2-yl)benzene.

In the crystal, the asymmetric unit contains two independent 1,4-bis(benzimidazol-2-yl)benzene molecules. The bond lengths are similar with those in literature (Bei *et al.*, 2000; Dudd *et al.*, 2003; Wu & Hu, 2009; Su *et al.*, 2011). The angles between benzimidazole rings (r.m.s. deviations of 0.0028 Å for molecule contain N1 and 0.0140 Å for molecule contain N3) and benzene rings (r.m.s. deviations of 0.0140 Å for C8–C13) are 16.8 and 14.2°. In the other molecule, the angles between benzimidazole rings (r.m.s. deviations of 0.0065 Å for molecule contain N5 and 0.0127 Å for molecule contain N7) and benzene rings (r.m.s. deviations of 0.0045 Å for C28–C33) are 26.1 and 37.3°. These angles are different with those reported by other researchers (31.0°, Bei *et al.*, 2000; Dudd *et al.*, 2003; 9.1°, Wu & Hu, 2009; 24.0° and 11. 6°, Su *et al.*, 2011). There are five kinds of hydrogen bonds which result in one dimensional network as that shown in Fig. 2 and Table 1.

### 2. Experimental

1,4-Bis(benzimidazol-2-yl)benzene was synthesized according to literature method (Alcalde *et al.*, 1992; Zhao *et al.*, 2012) and single crystals suitable for X-ray diffraction were obtained by slow evaporation of DMF solution at room temperature.

### 3. Refinement

N-bound H atoms were located in a difference Fourier map and were refined with bond-length restraints of N—H = 0.86 (2) Å. C-bound H atoms were positioned geometrically and treated as riding atoms with C—H = 0.93 Å, and with *U*_iso_(H) = 1.2*U*_eq_(C). Rigid-bond restraints (*DELU*) were applied for atoms C11 and C14.

### Crystal data

**Table:**

C_20_H_14_N_4_	*F*(000) = 1296
*M**_r_* = 310.35	*D*_x_ = 1.331 Mg m^−^^3^
Monoclinic, *P*2_1_/*c*	Mo *K*α radiation, λ = 0.71073 Å
Hall symbol: -P 2ybc	Cell parameters from 1697 reflections
*a* = 16.196 (3) Å	θ = 2.4–22.0°
*b* = 20.174 (3) Å	µ = 0.08 mm^−^^1^
*c* = 9.9010 (16) Å	*T* = 296 K
β = 106.733 (3)°	Block, yellow
*V* = 3098.1 (8) Å^3^	0.26 × 0.22 × 0.17 mm
*Z* = 8	

### Data collection

**Table:**

Bruker APEXII CCD diffractometer	5451 independent reflections
Radiation source: fine-focus sealed tube	3197 reflections with *I* > 2σ(*I*)
Graphite monochromator	*R*_int_ = 0.062
φ and ω scans	θ_max_ = 25.0°, θ_min_ = 2.0°
Absorption correction: multi-scan (*SADABS*; Bruker, 2001)	*h* = −16→19
*T*_min_ = 0.979, *T*_max_ = 0.986	*k* = −23→23
15697 measured reflections	*l* = −11→8

### Refinement

**Table:**

Refinement on *F*^2^	Secondary atom site location: difference Fourier map
Least-squares matrix: full	Hydrogen site location: inferred from neighbouring sites
*R*[*F*^2^ > 2σ(*F*^2^)] = 0.053	H atoms treated by a mixture of independent and constrained refinement
*wR*(*F*^2^) = 0.126	*w* = 1/[σ^2^(*F*_o_^2^) + (0.042*P*)^2^] where *P* = (*F*_o_^2^ + 2*F*_c_^2^)/3
*S* = 1.02	(Δ/σ)_max_ < 0.001
5451 reflections	Δρ_max_ = 0.21 e Å^−^^3^
450 parameters	Δρ_min_ = −0.21 e Å^−^^3^
5 restraints	Extinction correction: *SHELXL97* (Sheldrick, 2008), Fc^*^=kFc[1+0.001xFc^2^λ^3^/sin(2θ)]^-1/4^
Primary atom site location: structure-invariant direct methods	Extinction coefficient: 0.0018 (3)

### Special details

**Table:**

Geometry. All e.s.d.'s (except the e.s.d. in the dihedral angle between two l.s. planes) are estimated using the full covariance matrix. The cell e.s.d.'s are taken into account individually in the estimation of e.s.d.'s in distances, angles and torsion angles; correlations between e.s.d.'s in cell parameters are only used when they are defined by crystal symmetry. An approximate (isotropic) treatment of cell e.s.d.'s is used for estimating e.s.d.'s involving l.s. planes.
Refinement. Refinement of *F*^2^ against ALL reflections. The weighted *R*-factor *wR* and goodness of fit *S* are based on *F*^2^, conventional *R*-factors *R* are based on *F*, with *F* set to zero for negative *F*^2^. The threshold expression of *F*^2^ > σ(*F*^2^) is used only for calculating *R*-factors(gt) *etc*. and is not relevant to the choice of reflections for refinement. *R*-factors based on *F*^2^ are statistically about twice as large as those based on *F*, and *R*- factors based on ALL data will be even larger.

### Fractional atomic coordinates and isotropic or equivalent isotropic displacement parameters (Å^2^)

**Table:**

	*x*	*y*	*z*	*U*_iso_*/*U*_eq_	
N1	0.29247 (12)	−0.04939 (9)	0.8970 (2)	0.0422 (5)	
N2	0.27746 (13)	−0.04637 (9)	0.6655 (2)	0.0449 (5)	
N3	0.07524 (11)	0.26679 (8)	0.8390 (2)	0.0400 (5)	
N4	0.06709 (12)	0.27094 (9)	0.6109 (2)	0.0409 (5)	
N5	0.21368 (12)	−0.03614 (9)	0.3559 (2)	0.0457 (5)	
N6	0.25379 (13)	−0.06306 (9)	0.1650 (2)	0.0447 (5)	
N7	0.38516 (11)	0.29079 (8)	0.3466 (2)	0.0429 (5)	
N8	0.39950 (13)	0.27194 (9)	0.1317 (2)	0.0430 (5)	
C1	0.32991 (14)	−0.10557 (10)	0.8589 (3)	0.0419 (6)	
C2	0.37149 (15)	−0.15816 (11)	0.9408 (3)	0.0523 (7)	
H2A	0.3776	−0.1599	1.0371	0.063*	
C3	0.40316 (16)	−0.20745 (12)	0.8745 (3)	0.0572 (7)	
H3	0.4315	−0.2432	0.9273	0.069*	
C4	0.39416 (16)	−0.20559 (12)	0.7306 (3)	0.0585 (7)	
H4A	0.4166	−0.2401	0.6897	0.070*	
C5	0.35302 (15)	−0.15420 (12)	0.6475 (3)	0.0551 (7)	
H5	0.3469	−0.1529	0.5512	0.066*	
C6	0.32114 (14)	−0.10441 (10)	0.7147 (3)	0.0416 (6)	
C7	0.26227 (14)	−0.01539 (10)	0.7780 (3)	0.0404 (6)	
C8	0.22036 (14)	0.04919 (10)	0.7669 (2)	0.0380 (6)	
C9	0.18877 (15)	0.07285 (10)	0.8733 (3)	0.0463 (6)	
H9	0.1941	0.0470	0.9531	0.056*	
C10	0.14979 (15)	0.13358 (11)	0.8634 (3)	0.0483 (6)	
H10	0.1300	0.1486	0.9372	0.058*	
C11	0.13940 (13)	0.17303 (10)	0.7452 (2)	0.0366 (5)	
C12	0.17413 (15)	0.15063 (11)	0.6412 (3)	0.0504 (7)	
H12	0.1704	0.1770	0.5627	0.061*	
C13	0.21410 (16)	0.08991 (12)	0.6522 (3)	0.0549 (7)	
H13	0.2373	0.0760	0.5813	0.066*	
C14	0.09418 (14)	0.23666 (10)	0.7334 (2)	0.0379 (6)	
C15	0.03436 (14)	0.32525 (11)	0.7812 (3)	0.0425 (6)	
C16	0.00158 (16)	0.37657 (12)	0.8445 (3)	0.0597 (7)	
H16	0.0040	0.3748	0.9395	0.072*	
C17	−0.03427 (19)	0.42969 (13)	0.7627 (4)	0.0739 (9)	
H17	−0.0555	0.4650	0.8032	0.089*	
C18	−0.03965 (18)	0.43187 (13)	0.6192 (4)	0.0740 (9)	
H18	−0.0648	0.4686	0.5665	0.089*	
C19	−0.00906 (15)	0.38159 (12)	0.5539 (3)	0.0579 (7)	
H19	−0.0133	0.3830	0.4582	0.069*	
C20	0.02871 (14)	0.32833 (11)	0.6384 (3)	0.0428 (6)	
C21	0.19204 (14)	−0.10180 (11)	0.3204 (3)	0.0441 (6)	
C22	0.15135 (16)	−0.14810 (12)	0.3834 (3)	0.0582 (7)	
H22	0.1335	−0.1371	0.4619	0.070*	
C23	0.13831 (17)	−0.21025 (13)	0.3267 (3)	0.0627 (8)	
H23	0.1110	−0.2419	0.3670	0.075*	
C24	0.16497 (17)	−0.22720 (12)	0.2103 (3)	0.0637 (8)	
H24	0.1561	−0.2703	0.1756	0.076*	
C25	0.20405 (16)	−0.18225 (11)	0.1449 (3)	0.0587 (7)	
H25	0.2210	−0.1936	0.0658	0.070*	
C26	0.21713 (14)	−0.11937 (11)	0.2019 (3)	0.0440 (6)	
C27	0.24929 (14)	−0.01533 (10)	0.2595 (2)	0.0396 (6)	
C28	0.28177 (14)	0.05166 (10)	0.2531 (2)	0.0371 (6)	
C29	0.34685 (14)	0.06484 (10)	0.1920 (3)	0.0447 (6)	
H29	0.3704	0.0302	0.1531	0.054*	
C30	0.37728 (15)	0.12830 (11)	0.1877 (3)	0.0458 (6)	
H30	0.4212	0.1362	0.1466	0.055*	
C31	0.34253 (14)	0.18036 (10)	0.2446 (2)	0.0372 (5)	
C32	0.27666 (14)	0.16773 (10)	0.3045 (2)	0.0428 (6)	
H32	0.2529	0.2024	0.3427	0.051*	
C33	0.24615 (14)	0.10442 (10)	0.3080 (2)	0.0427 (6)	
H33	0.2013	0.0967	0.3473	0.051*	
C34	0.37521 (14)	0.24779 (10)	0.2431 (3)	0.0391 (6)	
C35	0.41685 (14)	0.34739 (10)	0.2989 (3)	0.0400 (6)	
C36	0.43613 (14)	0.40958 (11)	0.3605 (3)	0.0493 (6)	
H36	0.4292	0.4183	0.4488	0.059*	
C37	0.46551 (16)	0.45757 (11)	0.2884 (3)	0.0559 (7)	
H37	0.4784	0.4995	0.3281	0.067*	
C38	0.47638 (18)	0.44489 (13)	0.1577 (3)	0.0677 (8)	
H38	0.4973	0.4783	0.1118	0.081*	
C39	0.45718 (16)	0.38443 (12)	0.0935 (3)	0.0606 (7)	
H39	0.4645	0.3762	0.0052	0.073*	
C40	0.42666 (14)	0.33628 (10)	0.1652 (3)	0.0412 (6)	
H2	0.2599 (15)	−0.0344 (12)	0.5728 (19)	0.069 (9)*	
H4	0.0751 (15)	0.2586 (10)	0.5247 (19)	0.059 (8)*	
H6	0.2687 (15)	−0.0543 (12)	0.084 (2)	0.070 (8)*	
H8	0.3954 (16)	0.2505 (11)	0.051 (2)	0.069 (9)*	

### Atomic displacement parameters (Å^2^)

**Table:**

	*U*^11^	*U*^22^	*U*^33^	*U*^12^	*U*^13^	*U*^23^
N1	0.0531 (12)	0.0402 (11)	0.0389 (13)	0.0030 (9)	0.0219 (11)	0.0027 (9)
N2	0.0578 (13)	0.0460 (12)	0.0346 (14)	0.0063 (10)	0.0192 (12)	−0.0028 (11)
N3	0.0488 (11)	0.0394 (11)	0.0365 (12)	0.0025 (9)	0.0195 (10)	−0.0018 (9)
N4	0.0485 (12)	0.0412 (11)	0.0381 (14)	0.0025 (9)	0.0204 (11)	−0.0002 (10)
N5	0.0574 (12)	0.0460 (12)	0.0421 (13)	−0.0083 (10)	0.0277 (11)	−0.0020 (10)
N6	0.0608 (13)	0.0405 (11)	0.0409 (14)	−0.0066 (10)	0.0277 (12)	−0.0020 (10)
N7	0.0562 (12)	0.0356 (10)	0.0433 (13)	−0.0040 (9)	0.0249 (11)	−0.0006 (9)
N8	0.0548 (12)	0.0417 (12)	0.0379 (14)	−0.0039 (9)	0.0217 (11)	−0.0011 (10)
C1	0.0443 (13)	0.0400 (13)	0.0443 (17)	0.0018 (11)	0.0175 (13)	−0.0019 (12)
C2	0.0614 (15)	0.0470 (15)	0.0520 (18)	0.0081 (13)	0.0218 (15)	0.0081 (13)
C3	0.0599 (16)	0.0444 (15)	0.069 (2)	0.0109 (13)	0.0207 (17)	0.0033 (14)
C4	0.0586 (16)	0.0531 (16)	0.066 (2)	0.0107 (13)	0.0209 (17)	−0.0101 (15)
C5	0.0618 (16)	0.0547 (16)	0.0511 (19)	0.0094 (13)	0.0197 (15)	−0.0104 (14)
C6	0.0454 (13)	0.0388 (13)	0.0437 (16)	0.0015 (11)	0.0178 (13)	−0.0022 (12)
C7	0.0473 (14)	0.0398 (13)	0.0382 (16)	−0.0010 (11)	0.0190 (13)	−0.0013 (12)
C8	0.0439 (13)	0.0386 (13)	0.0350 (15)	0.0021 (10)	0.0167 (12)	0.0023 (11)
C9	0.0632 (15)	0.0428 (14)	0.0396 (16)	0.0090 (12)	0.0257 (14)	0.0068 (11)
C10	0.0663 (16)	0.0468 (14)	0.0414 (16)	0.0093 (12)	0.0304 (14)	0.0006 (12)
C11	0.0411 (12)	0.0356 (12)	0.0361 (15)	0.0007 (10)	0.0161 (12)	0.0001 (11)
C12	0.0674 (16)	0.0507 (15)	0.0415 (17)	0.0156 (13)	0.0288 (15)	0.0092 (12)
C13	0.0757 (18)	0.0563 (16)	0.0413 (17)	0.0196 (14)	0.0305 (15)	0.0058 (13)
C14	0.0443 (13)	0.0396 (13)	0.0340 (15)	−0.0063 (11)	0.0177 (12)	0.0005 (11)
C15	0.0432 (13)	0.0404 (13)	0.0476 (17)	0.0009 (11)	0.0191 (13)	−0.0013 (12)
C16	0.0655 (17)	0.0596 (17)	0.062 (2)	0.0130 (14)	0.0310 (16)	−0.0032 (15)
C17	0.084 (2)	0.0598 (18)	0.083 (3)	0.0289 (16)	0.033 (2)	−0.0013 (18)
C18	0.081 (2)	0.0575 (18)	0.084 (3)	0.0277 (15)	0.026 (2)	0.0120 (17)
C19	0.0630 (16)	0.0552 (16)	0.059 (2)	0.0122 (13)	0.0232 (15)	0.0148 (14)
C20	0.0419 (13)	0.0395 (13)	0.0504 (18)	0.0022 (11)	0.0185 (13)	0.0022 (12)
C21	0.0498 (14)	0.0458 (14)	0.0390 (16)	−0.0079 (11)	0.0162 (13)	0.0009 (12)
C22	0.0701 (17)	0.0579 (16)	0.0534 (19)	−0.0156 (14)	0.0287 (15)	0.0026 (14)
C23	0.0642 (17)	0.0567 (17)	0.069 (2)	−0.0192 (14)	0.0215 (17)	0.0094 (15)
C24	0.0701 (18)	0.0433 (15)	0.081 (2)	−0.0116 (13)	0.0264 (18)	−0.0039 (15)
C25	0.0723 (17)	0.0469 (15)	0.064 (2)	−0.0089 (13)	0.0310 (16)	−0.0103 (14)
C26	0.0479 (14)	0.0420 (14)	0.0440 (17)	−0.0039 (11)	0.0164 (13)	0.0019 (12)
C27	0.0466 (14)	0.0399 (13)	0.0353 (15)	−0.0014 (11)	0.0166 (12)	−0.0011 (11)
C28	0.0436 (13)	0.0394 (13)	0.0331 (14)	0.0002 (10)	0.0189 (12)	0.0015 (11)
C29	0.0536 (14)	0.0385 (13)	0.0516 (17)	−0.0020 (11)	0.0304 (14)	−0.0060 (12)
C30	0.0518 (14)	0.0473 (14)	0.0487 (17)	−0.0041 (11)	0.0309 (13)	−0.0061 (12)
C31	0.0420 (13)	0.0383 (13)	0.0346 (15)	0.0028 (10)	0.0163 (12)	0.0023 (11)
C32	0.0497 (14)	0.0408 (13)	0.0448 (16)	0.0074 (11)	0.0245 (13)	0.0008 (11)
C33	0.0453 (13)	0.0430 (14)	0.0465 (16)	0.0001 (11)	0.0240 (13)	0.0009 (12)
C34	0.0441 (13)	0.0407 (13)	0.0363 (15)	0.0014 (11)	0.0174 (12)	0.0005 (12)
C35	0.0455 (13)	0.0384 (13)	0.0398 (16)	0.0005 (11)	0.0180 (12)	0.0021 (11)
C36	0.0610 (16)	0.0428 (14)	0.0475 (17)	−0.0050 (12)	0.0210 (14)	−0.0061 (12)
C37	0.0678 (17)	0.0432 (14)	0.059 (2)	−0.0135 (13)	0.0220 (16)	−0.0018 (14)
C38	0.094 (2)	0.0559 (17)	0.059 (2)	−0.0259 (15)	0.0307 (19)	0.0058 (15)
C39	0.0809 (19)	0.0634 (17)	0.0452 (18)	−0.0196 (15)	0.0305 (16)	−0.0002 (14)
C40	0.0458 (13)	0.0400 (13)	0.0415 (16)	−0.0051 (11)	0.0183 (13)	0.0008 (11)

### Geometric parameters (Å, º)

**Table:**

N1—C7	1.329 (3)	C15—C16	1.392 (3)
N1—C1	1.388 (3)	C16—C17	1.368 (3)
N2—H2	0.912 (17)	C16—H16	0.9300
N3—C14	1.319 (3)	C17—H17	0.9300
N3—C15	1.392 (3)	C18—C17	1.400 (4)
N4—C14	1.355 (3)	C18—H18	0.9300
N4—C20	1.378 (3)	C19—C18	1.370 (3)
N4—H4	0.933 (16)	C19—H19	0.9300
N5—C27	1.318 (3)	C20—C19	1.391 (3)
N5—C21	1.389 (3)	C20—C15	1.392 (3)
N6—C27	1.359 (3)	C21—C22	1.391 (3)
N6—C26	1.379 (3)	C21—C26	1.393 (3)
N6—H6	0.916 (16)	C22—C23	1.365 (3)
N7—C34	1.316 (3)	C22—H22	0.9300
N7—C35	1.389 (3)	C23—H23	0.9300
N8—C34	1.363 (3)	C24—C23	1.385 (4)
N8—C40	1.380 (3)	C24—H24	0.9300
N8—H8	0.898 (16)	C25—C24	1.370 (3)
C1—C2	1.386 (3)	C25—H25	0.9300
C2—C3	1.370 (3)	C26—C25	1.380 (3)
C2—H2A	0.9300	C28—C29	1.384 (3)
C3—C4	1.391 (4)	C28—C33	1.393 (3)
C3—H3	0.9300	C28—C27	1.458 (3)
C4—H4A	0.9300	C29—H29	0.9300
C5—C4	1.371 (3)	C30—C29	1.377 (3)
C5—H5	0.9300	C30—H30	0.9300
C6—N2	1.382 (3)	C31—C30	1.385 (3)
C6—C5	1.384 (3)	C31—C32	1.386 (3)
C6—C1	1.393 (3)	C32—H32	0.9300
C7—N2	1.360 (3)	C33—C32	1.373 (3)
C7—C8	1.459 (3)	C33—H33	0.9300
C8—C13	1.381 (3)	C34—C31	1.461 (3)
C9—C10	1.369 (3)	C35—C36	1.391 (3)
C9—C8	1.381 (3)	C35—C40	1.396 (3)
C9—H9	0.9300	C36—C37	1.367 (3)
C10—H10	0.9300	C36—H36	0.9300
C11—C12	1.384 (3)	C37—C38	1.379 (4)
C11—C10	1.385 (3)	C37—H37	0.9300
C11—C14	1.466 (3)	C38—H38	0.9300
C12—C13	1.375 (3)	C39—C38	1.369 (3)
C12—H12	0.9300	C39—H39	0.9300
C13—H13	0.9300	C40—C39	1.376 (3)
			
C7—N1—C1	104.99 (19)	C29—C30—H30	120.0
C7—N2—C6	107.2 (2)	C31—C30—H30	120.0
C7—N2—H2	128.7 (16)	N6—C26—C25	132.6 (2)
C6—N2—H2	123.9 (16)	N6—C26—C21	105.4 (2)
C14—N3—C15	104.86 (19)	C25—C26—C21	122.0 (2)
C14—N4—C20	107.5 (2)	N5—C27—N6	113.21 (19)
C14—N4—H4	126.0 (14)	N5—C27—C28	124.4 (2)
C20—N4—H4	126.5 (14)	N6—C27—C28	122.4 (2)
C27—N6—C26	106.80 (19)	C20—C15—N3	109.85 (19)
C27—N6—H6	122.9 (15)	C20—C15—C16	120.2 (2)
C26—N6—H6	129.2 (16)	N3—C15—C16	130.0 (2)
C34—N7—C35	104.58 (19)	C12—C13—C8	121.1 (2)
C34—N8—C40	106.7 (2)	C12—C13—H13	119.4
C34—N8—H8	126.0 (16)	C8—C13—H13	119.4
C40—N8—H8	127.2 (16)	C3—C2—C1	117.5 (2)
N1—C7—N2	112.4 (2)	C3—C2—H2A	121.3
N1—C7—C8	124.9 (2)	C1—C2—H2A	121.3
N2—C7—C8	122.6 (2)	C33—C32—C31	120.6 (2)
N7—C34—N8	113.25 (19)	C33—C32—H32	119.7
N7—C34—C31	124.7 (2)	C31—C32—H32	119.7
N8—C34—C31	122.0 (2)	C30—C29—C28	121.1 (2)
C12—C11—C10	117.7 (2)	C30—C29—H29	119.5
C12—C11—C14	121.8 (2)	C28—C29—H29	119.5
C10—C11—C14	120.5 (2)	C38—C39—C40	117.2 (2)
C27—N5—C21	104.58 (18)	C38—C39—H39	121.4
C10—C9—C8	121.3 (2)	C40—C39—H39	121.4
C10—C9—H9	119.4	C4—C5—C6	116.4 (2)
C8—C9—H9	119.4	C4—C5—H5	121.8
N4—C20—C19	132.5 (2)	C6—C5—H5	121.8
N4—C20—C15	105.2 (2)	C37—C36—C35	118.5 (2)
C19—C20—C15	122.3 (2)	C37—C36—H36	120.7
N2—C6—C5	132.0 (2)	C35—C36—H36	120.7
N2—C6—C1	105.44 (19)	C36—C37—C38	121.1 (2)
C5—C6—C1	122.6 (2)	C36—C37—H37	119.5
C9—C10—C11	121.1 (2)	C38—C37—H37	119.5
C9—C10—H10	119.5	C2—C3—C4	121.9 (2)
C11—C10—H10	119.5	C2—C3—H3	119.1
C29—C28—C33	118.47 (19)	C4—C3—H3	119.1
C29—C28—C27	121.76 (19)	C18—C19—C20	116.4 (3)
C33—C28—C27	119.76 (19)	C18—C19—H19	121.8
C2—C1—N1	130.0 (2)	C20—C19—H19	121.8
C2—C1—C6	120.1 (2)	C5—C4—C3	121.6 (2)
N1—C1—C6	109.9 (2)	C5—C4—H4A	119.2
C13—C8—C9	117.7 (2)	C3—C4—H4A	119.2
C13—C8—C7	121.0 (2)	C17—C16—C15	117.9 (3)
C9—C8—C7	121.2 (2)	C17—C16—H16	121.0
N7—C35—C36	130.6 (2)	C15—C16—H16	121.0
N7—C35—C40	110.15 (19)	C23—C22—C21	118.0 (2)
C36—C35—C40	119.2 (2)	C23—C22—H22	121.0
N3—C14—N4	112.65 (19)	C21—C22—H22	121.0
N3—C14—C11	124.4 (2)	C24—C25—C26	116.9 (2)
N4—C14—C11	122.9 (2)	C24—C25—H25	121.5
C13—C12—C11	121.0 (2)	C26—C25—H25	121.5
C13—C12—H12	119.5	C25—C24—C23	121.8 (2)
C11—C12—H12	119.5	C25—C24—H24	119.1
C32—C33—C28	120.6 (2)	C23—C24—H24	119.1
C32—C33—H33	119.7	C19—C18—C17	122.1 (3)
C28—C33—H33	119.7	C19—C18—H18	119.0
C30—C31—C32	119.19 (19)	C17—C18—H18	119.0
C30—C31—C34	120.74 (19)	C22—C23—C24	121.4 (2)
C32—C31—C34	120.06 (19)	C22—C23—H23	119.3
N5—C21—C22	130.1 (2)	C24—C23—H23	119.3
N5—C21—C26	110.01 (19)	C39—C38—C37	121.8 (2)
C22—C21—C26	119.9 (2)	C39—C38—H38	119.1
C39—C40—N8	132.6 (2)	C37—C38—H38	119.1
C39—C40—C35	122.1 (2)	C16—C17—C18	121.2 (3)
N8—C40—C35	105.26 (19)	C16—C17—H17	119.4
C29—C30—C31	120.1 (2)	C18—C17—H17	119.4
			
C1—N1—C7—N2	0.4 (3)	C27—N6—C26—C21	−0.1 (3)
C1—N1—C7—C8	−177.3 (2)	N5—C21—C26—N6	−0.5 (3)
C35—N7—C34—N8	1.0 (3)	C22—C21—C26—N6	179.2 (2)
C35—N7—C34—C31	−179.7 (2)	N5—C21—C26—C25	179.3 (2)
C40—N8—C34—N7	−0.7 (3)	C22—C21—C26—C25	−0.9 (4)
C40—N8—C34—C31	179.96 (19)	C21—N5—C27—N6	−1.1 (3)
C14—N4—C20—C19	179.1 (2)	C21—N5—C27—C28	179.4 (2)
C14—N4—C20—C15	−0.4 (2)	C26—N6—C27—N5	0.8 (3)
C8—C9—C10—C11	1.1 (4)	C26—N6—C27—C28	−179.7 (2)
C12—C11—C10—C9	−3.6 (4)	C29—C28—C27—N5	153.5 (2)
C14—C11—C10—C9	177.3 (2)	C33—C28—C27—N5	−27.4 (3)
C7—N1—C1—C2	179.8 (2)	C29—C28—C27—N6	−26.0 (3)
C7—N1—C1—C6	−0.2 (2)	C33—C28—C27—N6	153.2 (2)
N2—C6—C1—C2	179.97 (19)	N4—C20—C15—N3	−0.2 (2)
C5—C6—C1—C2	−0.4 (4)	C19—C20—C15—N3	−179.8 (2)
N2—C6—C1—N1	−0.1 (2)	N4—C20—C15—C16	179.6 (2)
C5—C6—C1—N1	179.6 (2)	C19—C20—C15—C16	0.0 (4)
C10—C9—C8—C13	2.3 (4)	C14—N3—C15—C20	0.8 (2)
C10—C9—C8—C7	179.7 (2)	C14—N3—C15—C16	−179.0 (2)
N1—C7—C8—C13	160.4 (2)	C11—C12—C13—C8	0.4 (4)
N2—C7—C8—C13	−17.1 (3)	C9—C8—C13—C12	−3.0 (4)
N1—C7—C8—C9	−16.9 (3)	C7—C8—C13—C12	179.6 (2)
N2—C7—C8—C9	165.6 (2)	N1—C1—C2—C3	−179.6 (2)
C34—N7—C35—C36	177.0 (2)	C6—C1—C2—C3	0.4 (3)
C34—N7—C35—C40	−0.9 (3)	C28—C33—C32—C31	1.0 (3)
N1—C7—N2—C6	−0.4 (3)	C30—C31—C32—C33	0.1 (3)
C8—C7—N2—C6	177.33 (19)	C34—C31—C32—C33	−179.2 (2)
C5—C6—N2—C7	−179.4 (2)	C31—C30—C29—C28	−0.3 (4)
C1—C6—N2—C7	0.3 (2)	C33—C28—C29—C30	1.3 (3)
C15—N3—C14—N4	−1.1 (2)	C27—C28—C29—C30	−179.5 (2)
C15—N3—C14—C11	179.01 (19)	N8—C40—C39—C38	178.1 (2)
C20—N4—C14—N3	1.0 (3)	C35—C40—C39—C38	−1.2 (4)
C20—N4—C14—C11	−179.10 (19)	N2—C6—C5—C4	179.7 (2)
C12—C11—C14—N3	−165.6 (2)	C1—C6—C5—C4	0.1 (4)
C10—C11—C14—N3	13.4 (3)	N7—C35—C36—C37	−178.8 (2)
C12—C11—C14—N4	14.5 (3)	C40—C35—C36—C37	−1.0 (3)
C10—C11—C14—N4	−166.5 (2)	C35—C36—C37—C38	−0.3 (4)
C10—C11—C12—C13	2.9 (4)	C1—C2—C3—C4	−0.2 (4)
C14—C11—C12—C13	−178.1 (2)	N4—C20—C19—C18	−178.6 (2)
C29—C28—C33—C32	−1.7 (3)	C15—C20—C19—C18	0.9 (4)
C27—C28—C33—C32	179.2 (2)	C6—C5—C4—C3	0.1 (4)
N7—C34—C31—C30	−141.3 (2)	C2—C3—C4—C5	0.0 (4)
N8—C34—C31—C30	38.0 (3)	C20—C15—C16—C17	−1.2 (4)
N7—C34—C31—C32	38.0 (3)	N3—C15—C16—C17	178.6 (2)
N8—C34—C31—C32	−142.7 (2)	N5—C21—C22—C23	−179.6 (2)
C27—N5—C21—C22	−178.7 (3)	C26—C21—C22—C23	0.8 (4)
C27—N5—C21—C26	1.0 (3)	N6—C26—C25—C24	179.8 (2)
C34—N8—C40—C39	−179.4 (3)	C21—C26—C25—C24	0.0 (4)
C34—N8—C40—C35	0.0 (3)	C26—C25—C24—C23	1.1 (4)
N7—C35—C40—C39	−180.0 (2)	C20—C19—C18—C17	−0.7 (4)
C36—C35—C40—C39	1.8 (4)	C21—C22—C23—C24	0.3 (4)
N7—C35—C40—N8	0.6 (3)	C25—C24—C23—C22	−1.3 (5)
C36—C35—C40—N8	−177.6 (2)	C40—C39—C38—C37	−0.2 (4)
C32—C31—C30—C29	−0.5 (3)	C36—C37—C38—C39	1.0 (5)
C34—C31—C30—C29	178.9 (2)	C15—C16—C17—C18	1.4 (4)
C27—N6—C26—C25	−180.0 (3)	C19—C18—C17—C16	−0.4 (5)

### Hydrogen-bond geometry (Å, º)

**Table:**

*D*—H···*A*	*D*—H	H···*A*	*D*···*A*	*D*—H···*A*
N2—H2···N5	0.91 (2)	2.06 (2)	2.947 (3)	164 (2)
N4—H4···N3^i^	0.93 (2)	1.91 (2)	2.837 (3)	172 (2)
N6—H6···N1^ii^	0.92 (2)	2.00 (2)	2.910 (3)	171 (2)
N8—H8···N7^i^	0.90 (2)	2.15 (2)	3.041 (3)	174 (2)
C12—H12···N3^i^	0.93	2.57	3.396 (3)	148

Symmetry codes: (i) *x*, −*y*+1/2, *z*−1/2; (ii) *x*, *y*, *z*−1.
